# MUCO-DIS: a New AFM-Based Nanoscale Dissolution Technique

**DOI:** 10.1208/s12249-020-01697-x

**Published:** 2020-05-17

**Authors:** Muhammad Usman Ghori, Jorabar Singh Nirwan, Taimoor Asim, Younes Chahid, Samia Farhaj, Zara Khizer, Peter Timmins, Barbara R. Conway

**Affiliations:** 1 Department of Pharmacy, School of Applied Sciences, University of Huddersfield, Huddersfield, HD1 3DH UK; 2 School of Engineering, Robert Gordon University, Aberdeen, AB10 7GJ UK; 3 EPSRC Future Metrology Hub, School of Computing and Engineering, University of Huddersfield, Huddersfield, HD1 3DH UK

**Keywords:** mucoadhesion, *Ex vivo*, nanoscale, nanodissolution, adhesion force, surface texture, 3D printing, gastrointestinal mucosa, surface roughness, drug release, matrix erosion, AFM

## Abstract

Mucoadhesion-based drug delivery systems have recently gained interest because of their bio-adhesion capability, which results in enhanced residence time leading to prolonged duration of action with the mucosal surface, potentially improving compliance and convenience. Mucoadhesion testing of these formulations is widely reported; however, this is technically challenging due to the absence of any standard methods and difficulty in conducting mucoadhesion, formulation-mucosal surface interaction, mucosal surface topography and drug release in a single experiment. As these measurements are currently conducted separately, on replicate formulations, results can often be subjective and difficult to correlate. Hence, the aim of the present study was to develop a new AFM-based single-entity *ex vivo* muco-dissolution (MUCO-DIS) technique to simultaneously evaluate mucoadhesion force, 3D surface topography, polymer dissolution and drug release characteristics. To demonstrate the potential of the current technique, the interactions between model pectin microparticles containing metformin HCl and a range of gastrointestinal mucosal surfaces (gastric, small intestine, large intestine and buccal) were studied. This novel system has not only successfully determined the mucoadhesion force, polymer dissolution and drug release information but has also highlighted the difference in microparticle performance with different mucosal targets. The current work has highlighted the potential of this newly developed MUCO-DIS system and we believe this will be a valuable tool for characterising these popular pharmaceutical formulations. This technique could also provide an opportunity to other scientific fields to evaluate materials, substrate behaviour and their interactions in their hydrated state at nanoscale with real-time chemical and surface mapping.

## INTRODUCTION

Mucoadhesive performance is considered an important attribute of polymers intended for many drug delivery and tissue engineering applications, resulting in an extended duration of contact with the mucosal surface. This has applications for the delivery of drugs, bioactive peptides and nutritional ingredients to specific sites in the body (*e.g.*, oral cavity, nose, eye and vagina) (1). Numerous studies have investigated the mucoadhesive potential of polymers (2–6). Various theories have been proposed including: (a) diffusion, (b) adsorption, (c) wetting, (d) electrostatic and (e) fracture theory (7).

Upon delivery, these formulations tend to hydrate and interact with the mucosal surface. However, under conditions when the amount of water or biological fluid is limited, for example in the buccal cavity, rectum, nose and vagina, these formulations might require time to attain adequate hydration (8,9). In these circumstances, assessment of the formulation-mucosal interaction, especially the mucoadhesive force, becomes relevant. However, there is no standard test method established for mucoadhesion, and subsequently the data attained can be subjective and challenging to compare due the different parameters used as measures of adhesion force. Additionally, results depend on the experimental conditions, for example applied force, duration of contact, polymer concentration, pH level and ionic strength of biological fluids. This can lead to conflicting conclusions; for example, the work carried out by Schmidgall and Hensel concluded that LM pectin is more mucoadhesive than high-methoxy (HM) pectin using chemical analysis (10). However, Thirawong *et al*. found using texture analysis that HM pectin discs were more mucoadhesive (11). These may both be true depending on the circumstances involved and the analysis employed.

There are many different methods used in practice to evaluate mucoadhesion that can be broadly classified into direct and indirect techniques (7). The methods which evaluate time or force required for formulation detachment from the mucosal tissue (texture analyser (11), modified balance (12), tensile stress tester (13) and atomic force microscopy (AFM) (9,14)), are direct measurement techniques. However, indirect measurement techniques are also used which monitor the interaction between the polymer and mucus layer (rheology (15), ellipsometry (16), colloidal gold staining method (17), falling liquid film method (18,19), mucin particle method and BIACORE (20)).

AFM is considered a powerful technique which can be used to study adhesion between two materials and can map surface texture properties at the nanometre scale, facilitating analysis of single particles (9,14,22–24). In many cases, it has been used to examine the amount of material sticking to surfaces. The colloidal probe approach developed by Ducker *et al*. has been widely used to evaluate adhesion forces (21). This approach involves attaching a particle of the material of interest, most commonly a sphere, to an AFM cantilever. The AFM is used in force-displacement mode to quantify the forces between the colloidal probe and substrate surface.

Although AFM is effective in measuring mucoadhesive ability, there remains a challenge in developing a technique to reflect what happens *in vivo*, in a dynamic capacity within a liquid system. As the polymer hydrates and swells, drug is released, and its properties change. Currently, there is no approach for measuring mucoadhesion that can provide information on the mucoadhesion forces, polymer dissolution and drug release dynamically for an individual system in a single set of experiment. Hence, this research aims to develop a hydrodynamically characterised novel flow-through cell (muco-dissolution cell) that could be used with AFM for dynamic, nanoscale, single-entity, in-liquid *ex vivo* analysis. The substrate surface topography was studied before and after the experiments to determine if there was any deposition of polymer on the mucosal surface. Mucoadhesive microparticles of pectin and metformin HCl (a water-soluble antidiabetic drug) were used as a model formulation.

## MATERIALS AND METHODS

### Materials

High-methoxy citrus pectin and metformin HCl were purchased from Sigma-Aldrich Chemical Co., UK, and Tokyo Chemical Industry Ltd., UK, respectively. Sulphuric acid, phenol, Span 80, hexane and light liquid paraffin (dynamic viscosity 25–80 mPa s at 20°C) were purchased from Fisher Scientific Ltd., UK. All the chemicals were of analytical grade and used as received without any further modification and/or purification.

### Methods

#### Preparation of Pectin Microparticles

The pectin-based metformin microparticles were prepared using a water in oil (w/o) emulsion solvent evaporation technique (25–28). Briefly, 0.75% w/v of metformin HCl was added to 1.5% w/v aqueous pectin solution and stirred at 50°C until the metformin dissolved. The solution was poured into 200 mL of light liquid paraffin containing 0.5% v/v/Span 80. The aqueous phase was emulsified into the oily phase by stirring at 80°C for 2.5 h until the aqueous phase had evaporated. Microparticles were collected and washed three times with hexane, filtered, dried for 2 h and stored in desiccators at room temperature until further investigation.

#### Characterisation of Microparticles

Drug loading was determined by dissolving 50 mg of microparticles in 0.2 M phosphate buffer under sonication for at least 1.5 h until microparticles were fully dissolved and analysed using UV spectroscopy at a wavelength of 233 nm. The metformin concentration was determined using a standard calibration curve (linearity (*R*^2^) 0.999, LOQ (limit of quantification) 2.11 μg/mL and LOD (limit of detection) 0.81 μg/mL). The surface morphology of the microparticles was studied using scanning electron microscopy (SEM) (Quanta FEG 250) (29).

#### Experimental Setup for In-liquid Single-Particle *Ex Vivo* Nanoscale Dissolution

**Fig. 1:**
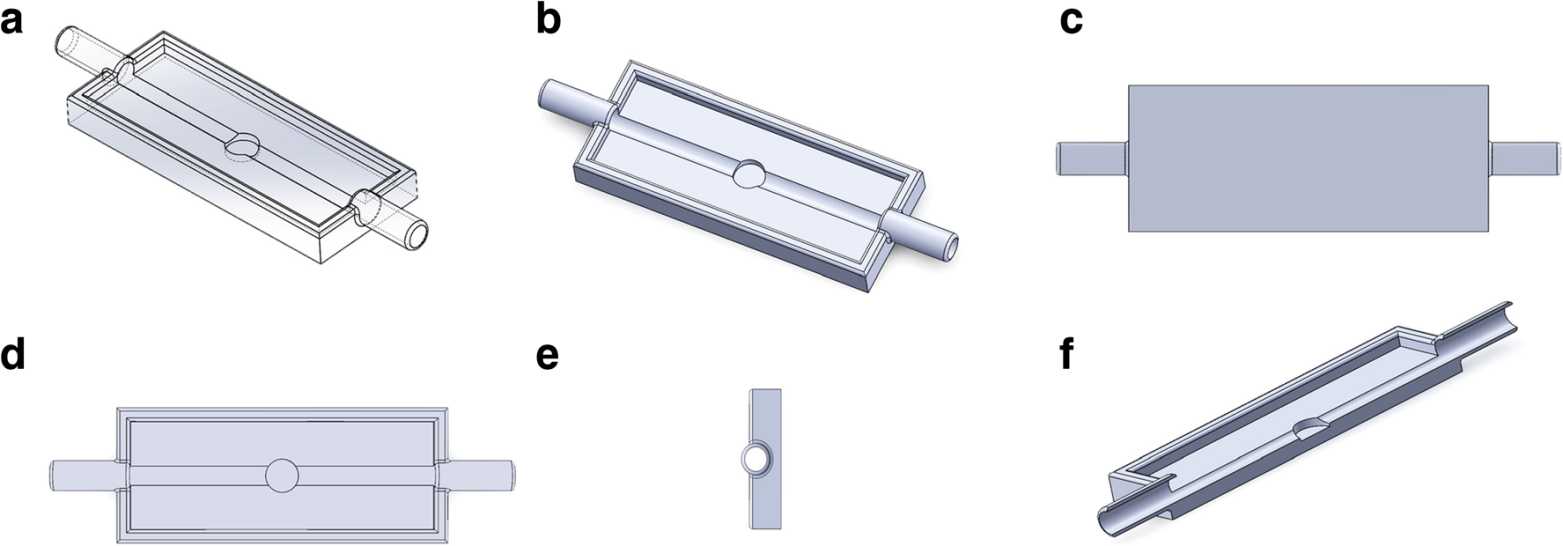
CAD (computer-aided design) model of muco-dissolution cell: **a**, **b** isometric, **c** bottom, **d** top, **e** side and **f** cross-sectional views of the model

The flow-through cell was developed using Solidworks® (version 25, 2017) and constructed from acrylonitrile butadiene styrene using a MakerBot Replicator™ 2X 3D printer (New York, USA) (Fig. 1). It was a rectangular shaped design with inlet and outlet ports intended for liquid loading and withdrawal, respectively. Both inlet and outlet ports were connected to a channel (of diameter 3.00 mm and depth 2.50 mm) which connected horizontally to a central sample compartment (10 mm) equipped with a magnetic wafer to facilitate the process of sample mounting and removal. The complete unit was connected to a peristaltic pump using silicon tubing (5-mm diameter), which had a port for sample loading and unloading. To understand liquid flow within the muco-dissolution cell, computational fluid dynamics (CFD) studies were carried out using ANSYS Fluent Software (ANSYS software Company, PA, USA). A mathematical computational approach was developed using three-dimensional Navier-Stokes equations.

#### Preparation of GI Tissues and Biological Fluids

Gastrointestinal (GI) mucosae from different parts of the porcine GI tract (buccal, stomach, small intestine and large intestinal tissues) were obtained from animals immediately after slaughter at a local abattoir. The tissues were washed thoroughly with deionised water to remove any undigested food and the underlying connective tissues were successively removed to isolate the mucosal membrane. Membranes were placed in normal saline solution at 4°C and were used within 4 h. The respective artificial biological media for the appropriate tissue (simulated gastric fluid, simulated intestinal fluid, simulated colonic fluid and artificial saliva) are described elsewhere (30). Moreover, the simulated fluids were used with their corresponding tissues and each fluid was subject to its own HPLC calibration.

#### 3D Surface Texture Analysis of GI Mucosa

Prior to muco-dissolution experiments, the tissue mucosa was positioned in the muco-dissolution cell under a steady stream of the appropriate simulated fluid flowing at 10 mL/min. After 30 min, surface topographical images of the GI mucosae were collected using AFM (Dimension Icon by Bruker, UK) in contact mode. A standard optical lever method with a small offset of force and low spring constant (0.07 N/m) bearing probes, MLCT (microlever cantilever tip), were employed. An auto-relocation algorithm was used to locate the same area post-dissolution. Quantitative surface texture parameters of the mucosal tissue before and after contact with the metformin-loaded pectin microparticles were determined using MATLAB®2017 software (The MathWorks, Inc., USA) (31,32).

#### In-liquid Mucoadhesion Force Measurement

**Fig. 2:**
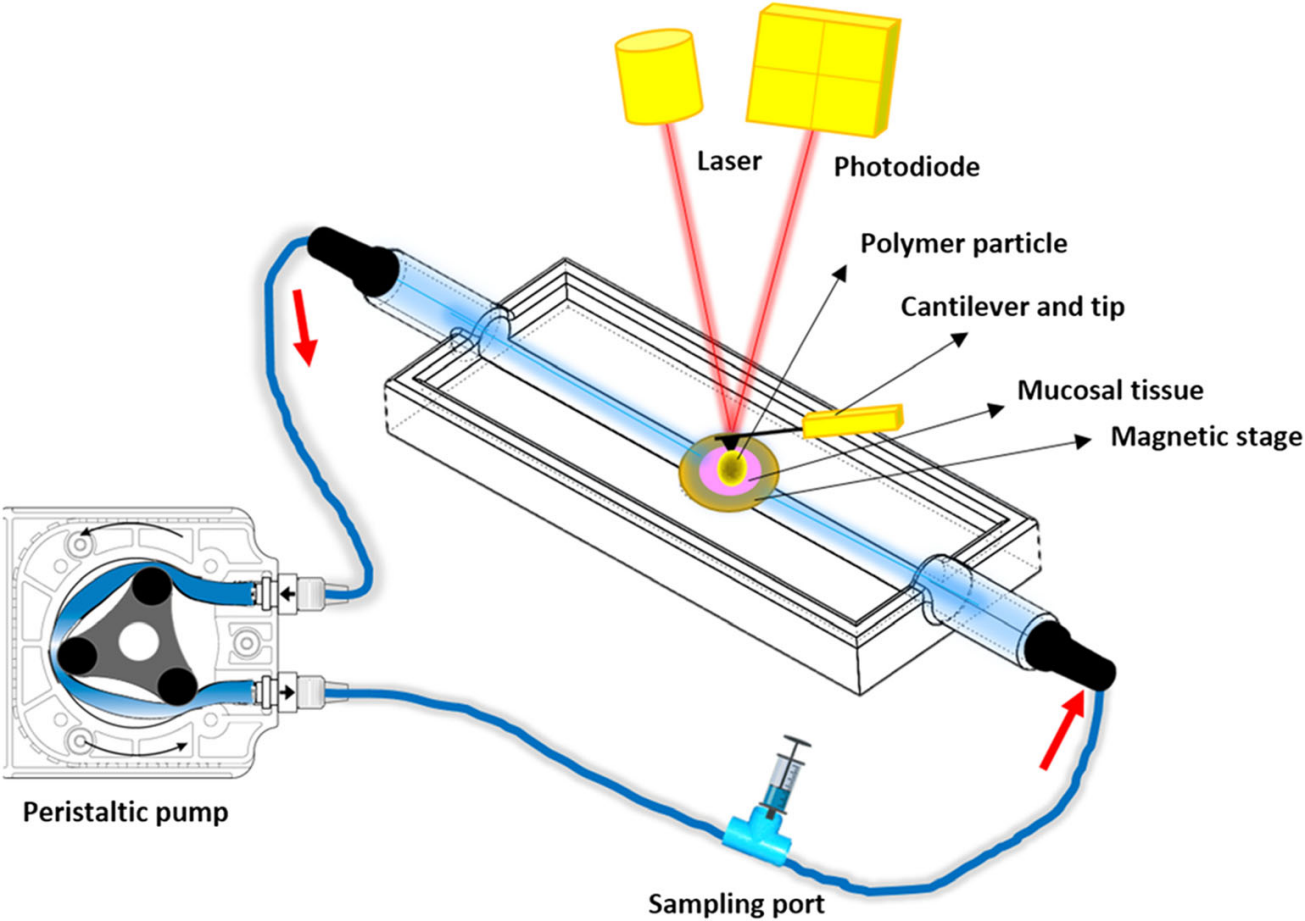
Schematic illustration of the experimental setup

**Fig. 3:**
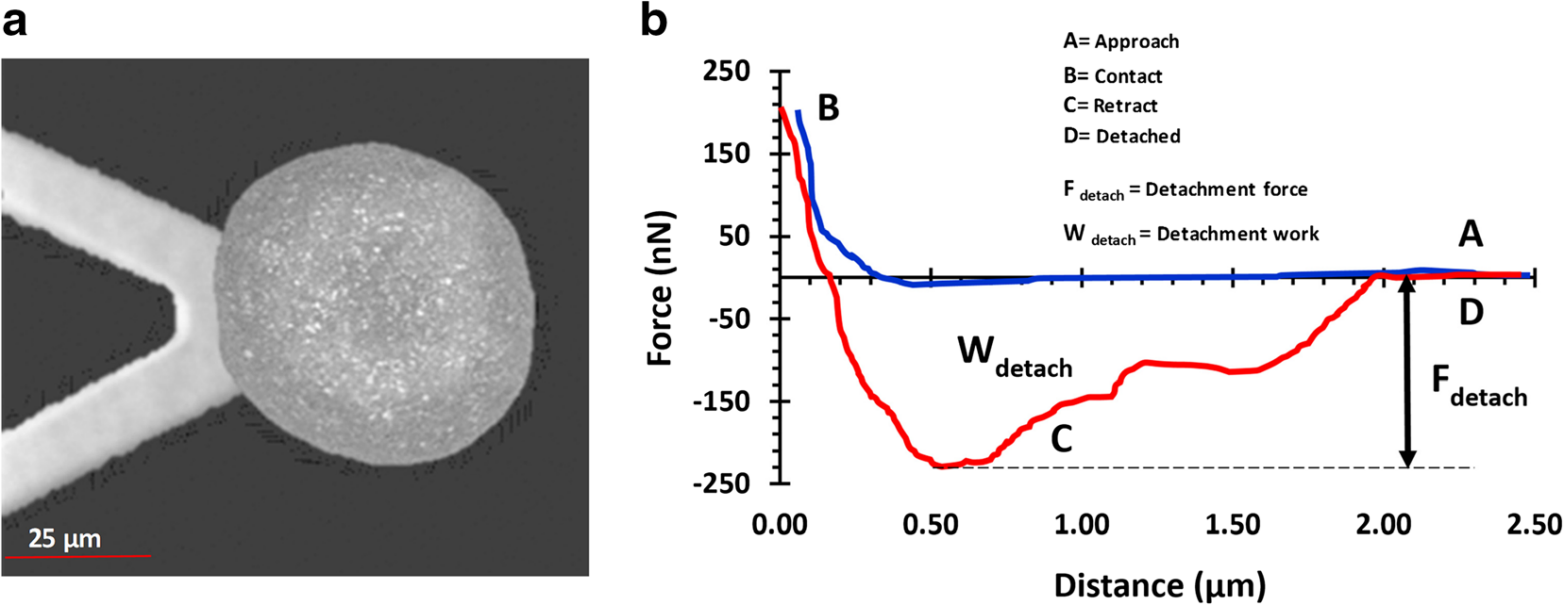
**a** SEM micrographs showing a microparticle attached a cantilever and **b** a typical force *vs*. distance curve acquired using AFM. The maximum depth of the peak is the force required to detach the AFM probe from the mucosal surface. This depicted curve is from the small intestinal mucosal (SIM) surface after 60-s holding time

In-liquid mucoadhesive force measurements were determined using the 3D-printed muco-dissolution flow-through cell attached to the atomic force microscope (Dimension Icon®, Bruker Nano Surfaces Ltd., Coventry, UK) in contact mode (Fig. 2). The experiments were performed at room temperature (25°C) and 40–50% relative humidity. Microparticles (40–150 μm) were carefully fixed to the colloidal probes using epoxy glue (fixation was confirmed using SEM, Fig. 3a). Before introducing the microparticle into the muco-dissolution cell, the appropriate biological medium was flushed through for at least 30 min at a flow rate of 10 mL/min removing any air bubbles from the tubing. Once a steady flow was established, the mucoadhesive force was determined 0, 5, 10, 20, 40, 60 and 120 s at fifteen various positions on tissues, and an average measurement was calculated and used for further data analysis. This necessitated the use of a different particle for each force measurement. A typical force *vs*. distance curve is illustrated in Fig. 3b. Moreover, to understand the impact of bio-adhesion on drug release properties, the same colloidal probe approach was used to evaluate the dissolution properties of microparticles in the absence of mucosal surfaces which acts as a control. After each predetermined time (0, 5, 10, 20, 40, 60 and 120 s), a 250-μL sample was collected from the sampling port using a microsyringe and an equal amount of fresh buffer was introduced. Surface texture analysis of the mucosal tissues post-experiment was carried out as detailed in the “3D Surface Texture Analysis of GI Mucosa” section.

#### Drug Release

Metformin concentrations were determined by HPLC using a reversed-phase C18 column (Phenomenex Ltd., Cheshire, UK) and mobile phase consisting of methanol and 0.05 mol L^−1^ (NH_4_)H_2_PO_4_ (35:65% v/v). The flow rate of the mobile phase was maintained at 1.5 mL/min with an injection volume of 20 μL. A wavelength of 234 nm was used for metformin detection and LCsolution software was used for data analysis. A linear relationship was established (*R*^2^ 0.999), and LOD and LOQ were 0.66 μg/mL and 2.01 μg/mL, respectively. (33).

#### Polymer Dissolution and Erosion Analysis

To quantify dissolved pectin, a previously described procedure was adapted (31,32,34,35). Briefly, 20 μL of 5% v/v phenol was added to 20 μL of the dissolution medium, containing any dissolved pectin, in microplate wells followed by mixing for 5 min using a shaking plate mixer. Concentrated H_2_SO_4_ (100 μL) was added to each well and mixed again for 5 min. The solutions were then incubated for 15 min at room temperature (20–25°C) before the UV absorbance was read at 490 nm using a microplate reader and dissolved pectin was quantified using a standard calibration curve. Additionally, the overall matrix erosion was calculated by simply summing the drug and pectin dissolved at each specified time point.

#### Statistical Analysis

SPSS software version 20 was used for two-way analysis of variance with post hoc test (*p* < 0.05).

## RESULTS AND DISCUSSION

**Fig. 4:**
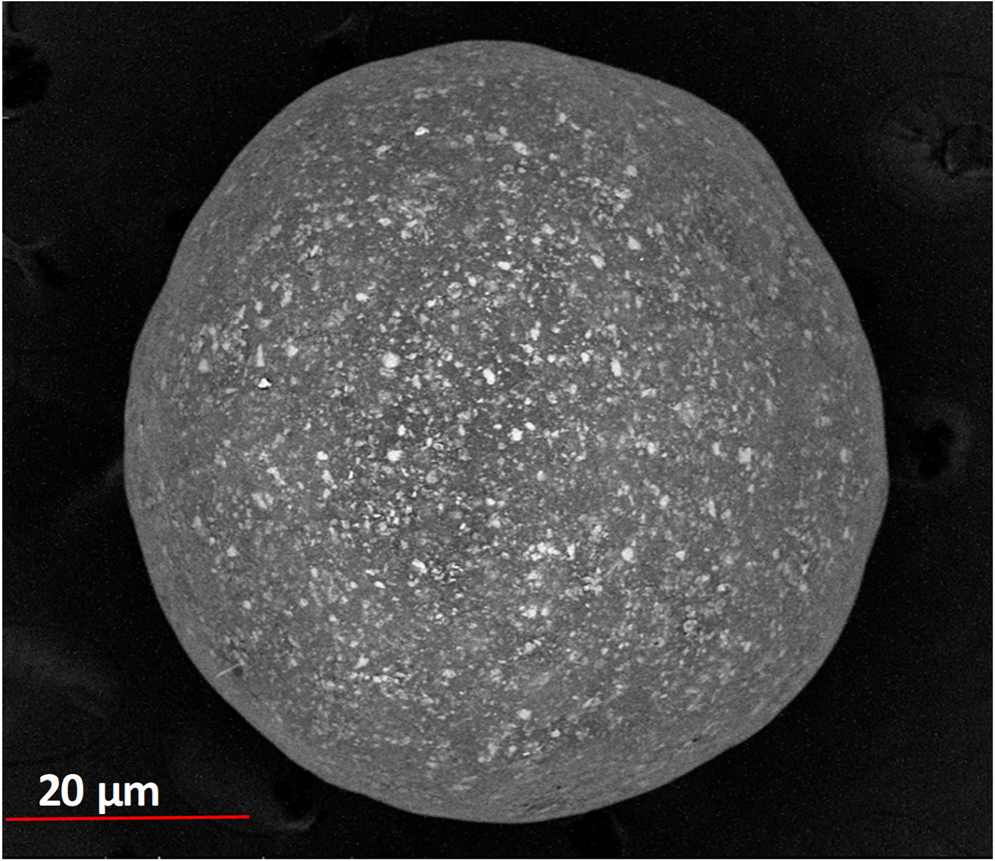
SEM micrograph of pectin-based metformin microparticle

Drug entrapment efficiency in the microparticles was 74.1 ± 4.2% which is in good agreement with the literature (25,26). The microparticles were spherical with no obvious cracks or holes on their surface (Fig. 4); hence, the quality was deemed suitable for the current muco-dissolution experiment.

### Hydrodynamics Studies of Muco-dissolution Cell

**Fig. 5:**
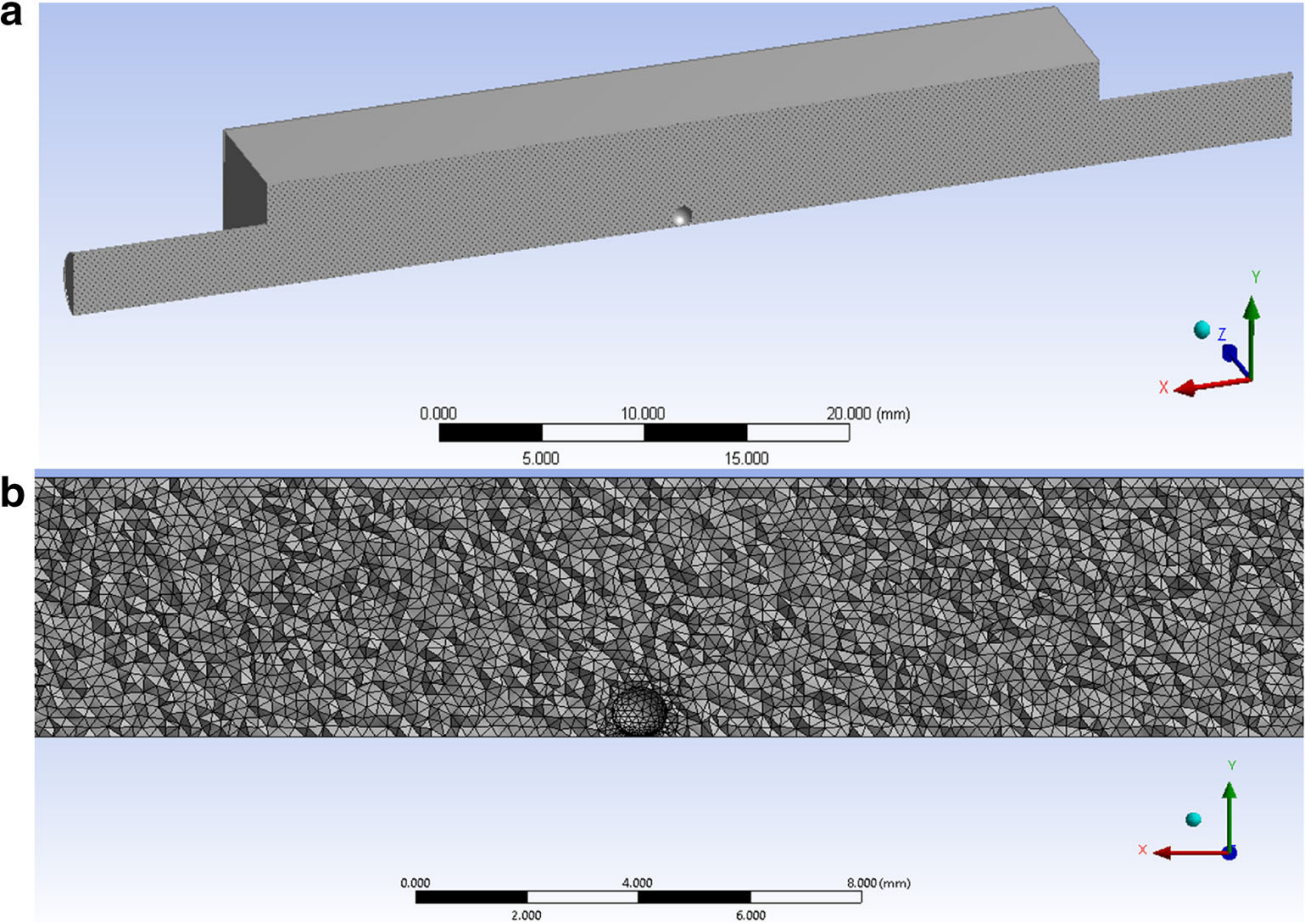
**a** Cross-sectional view of the apparatus and **b** meshing of the flow domain

**Fig. 6:**
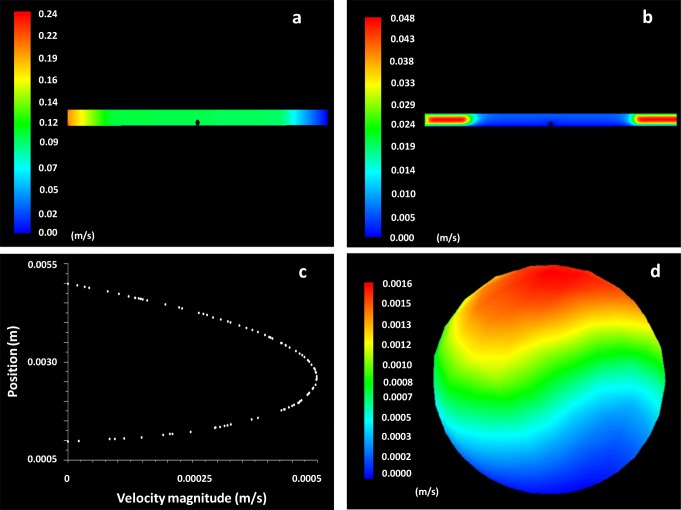
Variations in **a** static gauge pressure (in Pa), **b** flow velocity magnitude (in m/s), **c** velocity profile in the middle region of the within the flow domain, **c** velocity profile in the mid-section of muco-dissolution cell and **d** wall shear stress on the surface of the particle

Fused deposition modelling (FDM) was successfully used for the development of the muco-dissolution cell and computational fluid dynamics studies were used to understand liquid flow and mixing phenomena (ANSYS Fluent Software, ANSYS software Company, PA, USA). The numerical model of the apparatus is shown in Fig. 5a. The particle/sphere of radius 0.5 mm was placed in the central compartment of the cell and attached with a thin stem probe. An unstructured tetrahedral mesh was generated within the flow domain, as shown in Fig. 5b, with a mesh size that had previously been determined to be suitable for accurate prediction of the flow features within the apparatus (results not shown). Three-dimensional Navier-Stokes equations were numerically solved for the laminar flow of the media. Mass flow inlet boundary conditions were 1 mL/min, while the outlet boundary was kept at atmospheric pressure conditions. The numerical model was considered to be isothermal and a steady-state solver was employed to run the simulations in an iterative manner. The density and dynamic viscosity of water at room temperature were used, *i.e.* 998.2 kg/m^3^ and 0.890 cP. The flow domain was spatially discretised using the Green-Gauss node–based method, while using second-order upwind schemes for solving the pressure and momentum terms. A convergence criterion of 1 × 10^−6^ was used for mass and momentum conservation. The resulting flow fields were captured as shown in Fig. 6a, b. The contours have been shown on a cross-sectional plane in the *z*-direction. The flow is taking place from left to right in the contours. Figure 6a depicts the local variations in static gauge pressures within the flow domain. The static pressure within the muco-dissolution cell was 0.1 Pa. It is evident that the pressure change within the muco-dissolution cell is gradual, so AFM imaging is therefore possible. Additionally, Fig. 6b depicts the spatial variations in the flow velocity magnitude within the flow domain. A parabolic velocity profile is evident which has been further plotted in Fig. 6c. The hydrodynamic flow forces acting on the particle were 0.003 μN, − 0.008 μN and 1 × 10^−6^ μN in *x-*, *y-* and *z*-directions, respectively. The existence of parabolic flow indicates that the particle is under the action of flow forces. These forces exerted also suggested that the particle was under the action of a lift force, which is the primary force acting on it. The drag force acting on the particle is 64% less than the lift force, while the side force, as expected in the case of a sphere, is negligibly small compared with the other two force components. Another point of interest here is that in both *x*- and *y*-directions, the shear force acting on the particle is significantly higher than the pressure force. The average wall shear stress acting on the surface of the particle is 0.0004 Pa, while the maximum wall shear stress is 0.0017 Pa. Figure 6d depicts the local variations in the wall shear stress acting on the particle’s surface. It can be seen that the wall shear stress is maximal on the upper section of the particle, while it decreases in –ive *z*-direction, *i.e.* towards the base of the muco-dissolution cell. Overall, the CFD findings confirm a good mixing of liquid around the point of contact of microparticle.

### 3D Surface Texture Analysis Before Muco-dissolution Experiment

**Fig. 7:**
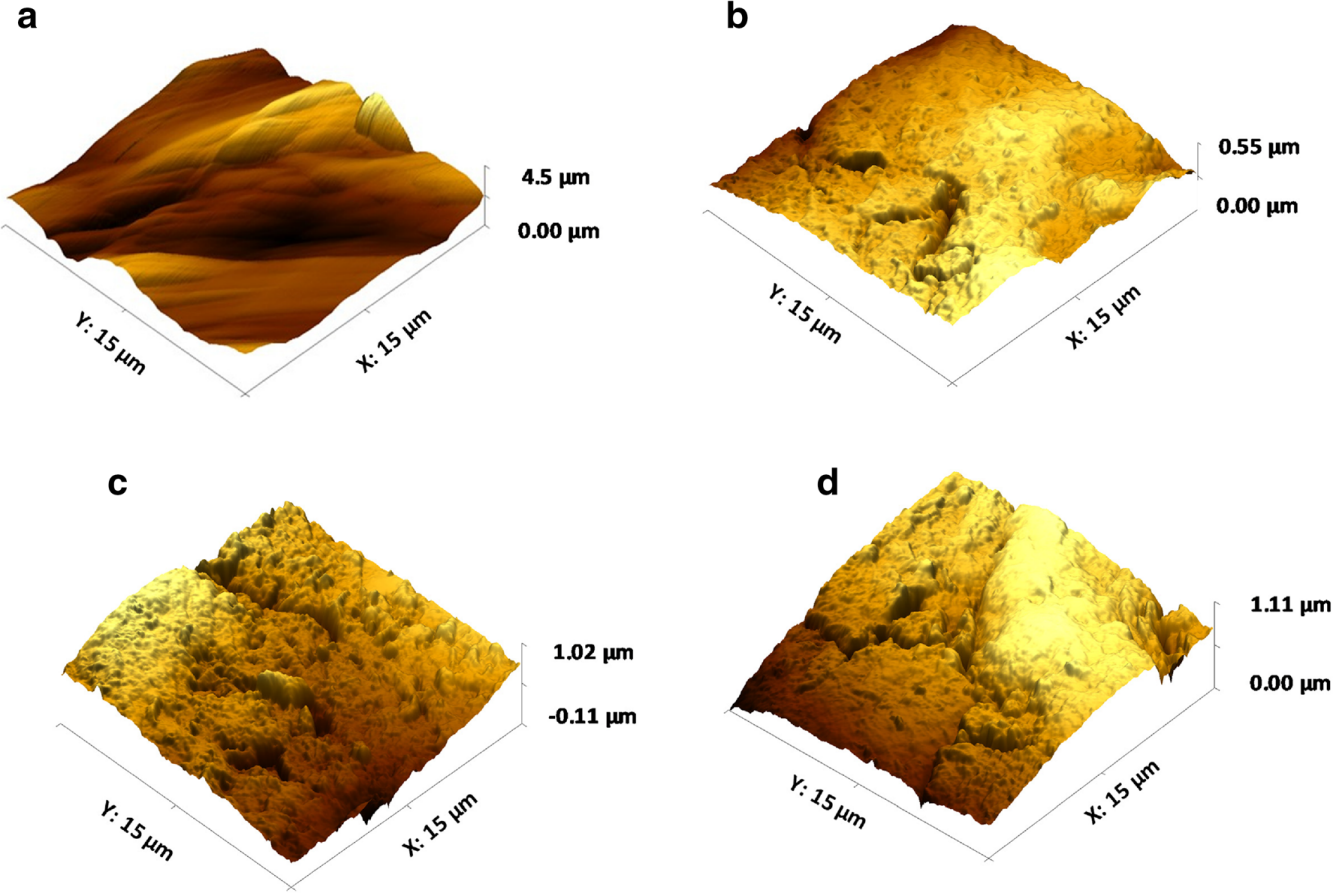
3D AFM images of **a** gastric, **b** small intestinal, **c** large intestinal and **d** buccal mucosal surfaces before the muco-dissolution experiment

3D surface texture analysis was conducted on the hydrated GI mucosal surfaces (Table I). The measured parameters relate to the arithmetical mean height (Sa), root mean square height (Sq), maximum peak height (Sp) and maximum valley depth (Sv) of the surfaces. As Sa and Sq are generally used to represent an overall measure of the roughness of the surface, the initial analysis of the mucosal surfaces before dissolution revealed the greatest surface roughness in the gastric mucosa (GM) followed by the small intestinal mucosa (SIM), large intestinal mucosa (LIM) and buccal mucosa (BM). Additionally, the Sp and Sv were high for GM and low for BM. However, although the surface roughness was greater for SIM than LIM, the height of the highest peak and the depth of the deepest valley were both larger for LIM. This may be due to the fact that Sa and Sq are not able to effectively differentiate valleys, peaks and the spacing of various surface texture parameters. This was also evident in the AFM images of the mucosal surfaces prior to dissolution (Fig. 7a–d). The surface of GM has multiple large peaks and valleys with no flat surface (Fig. 7a) whereas SIM, LIM and BM have a flatter surface although numerous valleys and smaller peaks were still visible (Fig. 7b, c and d, respectively). This was especially apparent in the AFM image of the LIM which shows deeper valleys and higher peaks compared with the SIM and BM (also confirmed by data in Table I).

**Table I:** 3D Surface Texture Parameters of GI Mucosal Surfaces Used in This Experiment (*n* = 5, Standard Deviations Are in Parenthesis)

Surface texture parameter	Microparticle surface	Mucosal surface before dissolution			
		GM	SIM	LIM	BM
Sa (μm)	20.11 (4.55)	123.01 (4.18)	100.2 (5.15)	94.5 (11.12)	77.8 (8.11)
Sq (μm)	24.19 (3.88)	152.6 (6.19)	123.6 (8.12)	131.2 (8.22)	92.7 (4.56)
Sp (μm)	90.44 (5.88)	335.4 (9.11)	289.63 (6.37)	301.3(15.33)	241.3 (6.59)
Sv (μm)	111.26(6.15)	652.8 (10..13)	555.2 (15.29)	591.4 (20.25)	509.6 (15.30)

*GM* gastric mucosa, *SIM* small intestinal mucosa, *LIM* large intestinal mucosa, *BM* buccal mucosa, *GI* gastrointestinal, *Sa* mean height, *Sq* root mean square height, *Sp* maximum peak height, *Sv* maximum valley depth

### Mucoadhesion Force and Work of Detachment Studies

**Fig. 8:**
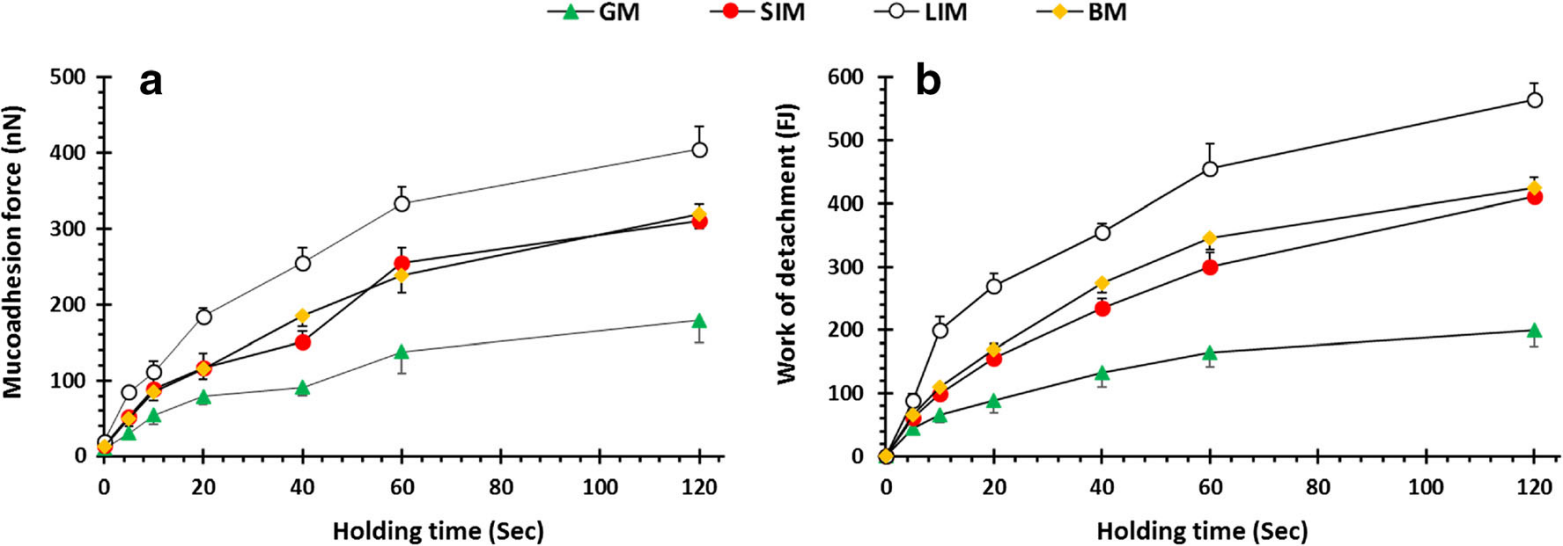
**a** Mucoadhesion force and **b** work of detachment as a function by holding time using newly developed muco-dissolution cell

Mucoadhesion force (nN) and work of detachment (FJ) with respect to holding time (s) are displayed in Fig. 8a and b, respectively. There was a statistically significant higher mucoadhesive force and work of detachment for LIM compared with the other mucosae. The lowest mucoadhesive force and work of detachment generated from the interaction with GM were also statistically significantly lower than for the other mucosal surfaces. There were statistically insignificant differences between the SIM and BM at all time points except for 40 s. This may be due to ionisation of the pectin at higher pH values of LIM, SIM and BM which causes it to form a hydrogel, enabling mechanical entanglement. This will increase the contact surface for hydrogen bonding to occur between the pectin and the mucosal surface (36). There was an increase in mucoadhesive force and work of detachment as the holding time was increased (Fig. 8a, b). This was expected and in agreement with previous findings which attributed this to an increase in chain entanglement and hydrogen bonding between the polymer network and mucosa due to increased hydration and swelling of the pectin surface as contact time is extended (11,22).

### Drug Release, Polymer Dissolution and Matrix Erosion Studies

**Fig. 9:**
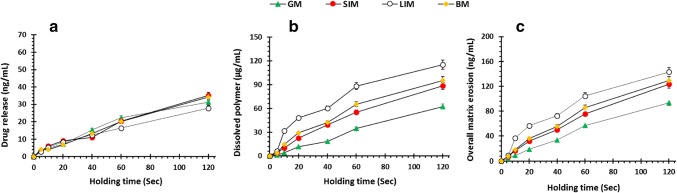
**a** Drug release, metformin HCl; **b** polymer dissolution, pectin; and **c** overall matrix erosion as a function by holding time by AFM studies using newly developed muco-dissolution (MUCO-DIS) cell in the presence of mucosal surfaces

Drug release, polymer dissolution and overall matrix erosion profiles were also obtained from the same formulations (Fig. 9a–c). Polymer located at the outermost surface of the formulation is in contact with the dissolution media, and over time the polymeric network starts to dissolve leading to disentanglement of the polymeric chain. This matrix system dissolves from the surface. The mechanism and rate of drug release are controlled by swelling, matrix erosion and/or diffusion of drug through the gel layer (32,34,37,38). Figure 9 a shows that drug release increases with time for all surfaces. Any differences in drug release between the different mucosal surfaces were statistically insignificant until after 60 s. Thereafter, drug release on the LIM was significantly slower possibly due to hydration and swelling of the pectin owing to the absence of villi enabling more interaction between the mucosal surface and the pectin. This is also why more polymers are dissolved on the LIM compared with the other mucosal surfaces and may account for the increase in dissolved polymer from 0- to 20-s holding time. Additionally, as the pKa of the pectin used in this study was 3.5 (39), it is possible that ionisation of the polymeric chains, higher pHs, led to greater polymer dissolution than for GM. Figure 9c displays the overall matrix erosion which is the collective amount of drug and polymer dissolved (31). There is an increase in overall matrix erosion as holding time is increased due to increased hydration and swelling of the polymer.

**Fig. 10:**
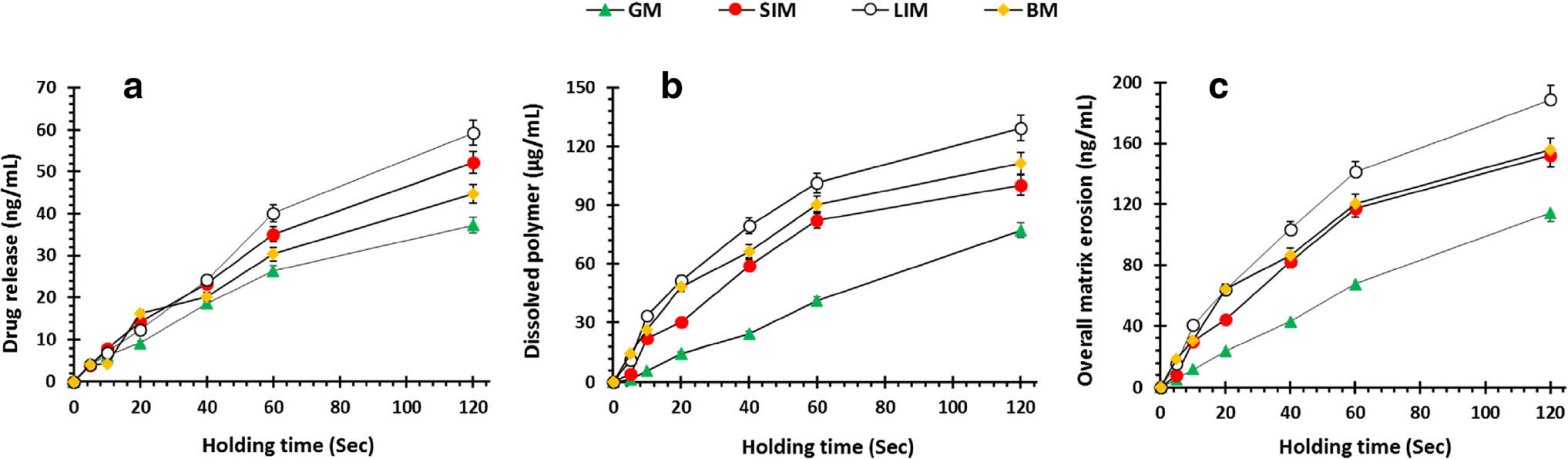
**a** Drug release, metformin HCl; **b** polymer dissolution, pectin; and **c** overall matrix erosion as a function by holding time by AFM studies using newly developed muco-dissolution (MUCO-DIS) cell in the absence of mucosal surfaces which acts as a control to understand the impact of bio-adhesion on drug release, polymer dissolution and overall matrix erosion

Furthermore, the drug release, polymer dissolution and overall matrix erosion in the absence of mucosal surfaces is displayed in Fig. 10a–c. This data revealed that the drug release from 60 s onwards was significantly greater in the simulated intestinal fluid followed by simulated colonic fluid, artificial saliva and gastric fluid (Fig. 10a). This supports the current understanding in the literature that metformin exhibits slower dissolution in lower pH media due to higher solvation and a larger hydrodynamic radius in the acidic media caused by additional protonation of metformin (40,41). Moreover, the ionisation of the polymeric chains at higher pH values produced similar polymer dissolution profiles as the profiles containing mucosal surfaces (Fig. 10b). However, the lack of mucoadhesion due to the absence of a mucosal surface resulted in insignificant differences in polymer dissolution between the simulated intestinal fluid and artificial saliva in the first 20 s. This was in contrast with the polymer dissolution profiles displayed in Fig. 9b in which polymer dissolution for LIM was significantly higher than the other mucosal surfaces due to the initial interaction between the polymer and LIM.

### 3D Surface Texture Analysis After Muco-dissolution Experiment

**Fig. 11:**
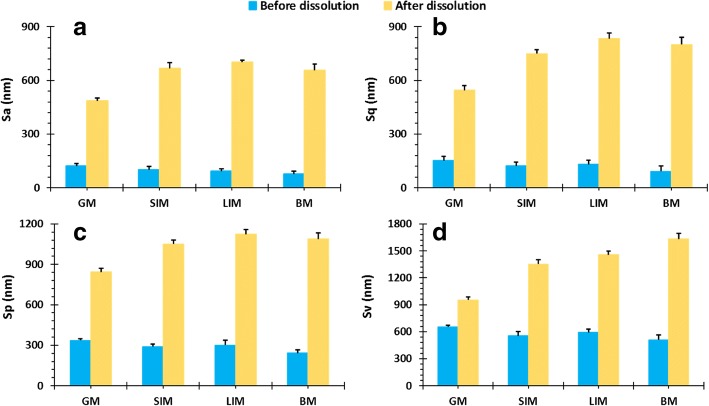
Comparison of 3D surface texture parameters of GI mucosal tissues before and after dissolution experiments

**Fig. 12:**
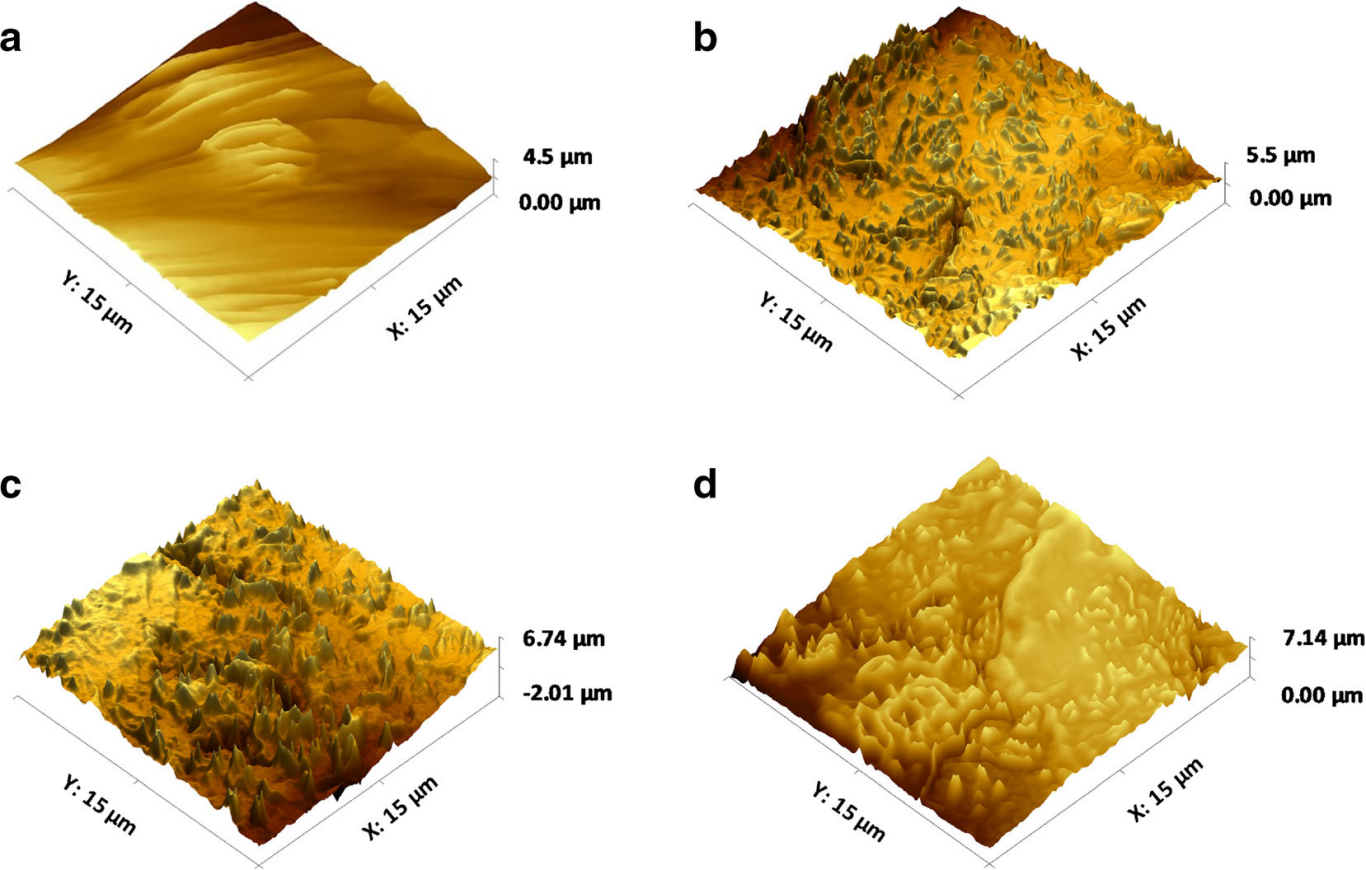
3D AFM images of **a** gastric, **b** small intestinal, **c** large intestinal and **d** buccal mucosal surfaces after the muco-dissolution experiment

Following dissolution, surface texture parameters for all mucosal surfaces increased due to interactions between the pectin and the mucosa (Fig. 11a–d). The differences in surface texture parameters between SIM, LIM and BM were statistically insignificant whereas they were significantly lower in the GM. AFM images of the mucosal surfaces after dissolution also confirmed this finding (Fig. 12a–d). The surface of the GM (Fig. 12a) is visibly smoother than the surfaces of the SIM, LIM and BM (Fig. 12b, c and d, respectively) and displays multiple ridges rather than large peaks or valleys. Conversely, the surfaces of the SIM, LIM and BM clearly display sizeable peaks and valleys, especially evident for the LIM. It is possible that this increase in surface roughness may be due to polymer deposition onto the surface of the mucosal membrane following ionisation of the polymeric chains (42). Moreover, at higher pH values, pectin is able to form a hydrogel which also contributes to formation of interchain bridges between the polymer and the mucosal surface (43,44).

A study conducted by Thirawong *et al*. investigating the mucoadhesive properties of various pectins on GI mucosa also found that adhesion was better when the pH value of the medium was 4.8 compared with pH 1.2. The authors concluded that this was due to pectin rapidly converting from carboxylate anions (pectin salt) to free carboxyl groups or unionised forms (pectinic acid), as the concentration of hydrogen ions increased at pH 1–2 resulting in a reduction in the swelling ability of the pectin upon hydration (11,22).

Differences in the anatomy and structure of the mucosal surfaces may also be responsible for the increased interactions between the pectin and mucosa. Variations in mucin content in different regions of the GI tract result in differences in mucoadhesive properties. Mucin released by goblet cells in the intestines forms a mucus layer which may develop a viscous gel covering the epithelia depending on the pH of the media (11). The presence of larger quantities of goblet cells in the large intestine also results in greater levels of mucin on the mucosa of the intestines (5). As discussed previously, the absence of villi in the LIM allows greater interactions between the mucosal surface and the pectin, thereby increasing mucoadhesiveness. It has been reported that dissociation of carboxyl groups of carbomer and electrostatic repulsion between these groups resulted in the uncoiling and extension of the molecule at high pH causing swelling and gel formation of carbomer (34). The enlargement of the swollen polymer and mucus enhanced the interdiffusion process and caused mechanical entanglement and an increase in surface contact for hydrogen bonding and/or electrostatic interactions between the polymer and the mucosal network.

## CONCLUSIONS AND FUTURE PROSPECTS

This study has demonstrated the successful development of a new in-liquid single-particle *ex vivo* nanoscale dissolution technique for mucoadhesive force, 3D surface topography, polymer dissolution, drug release and erosion analysis in a single experiment, negating the need for separate investigations. The CFD findings confirm a good mixing of liquid around the point of contact of microparticle. Furthermore, the present study demonstrated that the current system has a potential to be used for multiple GI target sites where mucoadhesion is playing a vital role in controlling the drug release. It is anticipated that this synchronised approach will provide researchers an opportunity to explore other polymers and newly developed mucoadhesive formulations in an environment that better reflects *in vivo* situation. Additionally, CFD findings revealed that this system can be adapted for use for other industrially relevant materials and substrates to understand their behaviour and interaction in hydrated state at a nanometric scale with chemical and surface mapping.
